# Editorial: Advances in host-pathogen interactions for diseases in animals and birds

**DOI:** 10.3389/fvets.2023.1282110

**Published:** 2023-09-11

**Authors:** Mrigendra Rajput, Neelu Thakur

**Affiliations:** Department of Biology, University of Dayton, Dayton, OH, United States

**Keywords:** host-virus interaction, virome, antiviral protein, endogenous viral elements, virus symbiosis

## Introduction

Host-virus interactions are complex cross-talk between the virus and its host cell. These interactions are not limited to causing disease or changing the physiology of host cells but they also influence viruses. Studies showed bidirectional changes in viruses and their host which co-evolved together (1, 2). The host body harbors numerous microbes, collectively it is called the microbiome (3, 4). However, the majority of microbiome studies are focused on bacterial populations, missing important viral components which is also known as virome (5). It is estimated that the number of viruses in the host is around ten times more than the host-inhabited bacterial population (6). Interestingly, virus or bacteriophages significantly influence bacterial population or their physiology in the host (7). Studies also indicated that virulence factors in bacteria are acquired from their bacteriophage (8–10). The overall influence of the virus on the host body and ecosystem is very vast and complex. But the current topic focuses on the interactions between vertebrate hosts like animals with their viruses.

## The majority of viruses are commensal in the host's body

The presence of virus in the host body does not always cause diseases. A majority of viruses present in the host body are commensal (11). Host body contains both DNA and RNA virus (12, 13), and these viruses can be localized on the skin (14), peripheral blood (15, 16), or internal organs such as the lungs, liver, spleen, kidney, or heart (17). The number and type of virus in the virome are greatly influenced by the environment, dietary practices, and their location in the body (18, 19). Despite their importance in shaping the overall health of the host, we have very limited information about our virome and its interactions with host cells. The majority of virome study is limited to metagenomic sequencing, which is trying the uncover the least explored part of virome or “viral dark matter” and its potential role in the evolution and shaping of the overall health and immune system of the host (20, 21).

## Virus and its symbiotic relation with its host

There are several pieces of evidence showed that the virus and its host can benefit each other. A study with tropical panic grass, fungal endophyte, and fungal virus showed a three-way symbiosis. Where virus-infected fungus confers heat tolerance to both, tropical panic grass and fungal endophyte. Virus-free fungi were unable to confer heat tolerance, but heat tolerance was restored after the virus is reintroduction to fungi (22). Similarly, a study with parasitoid wasp showed obligatory mutualism between virus and parasitoid wasps. Successful development of the parasitoid wasp's egg within the host depends on the presence of the virus which suppresses the host's immune response (23).

Herpesvirus are important virus family which infect humans and animals and they could cause lifelong latency in the host (24–26). It is found that herpesvirus latency provides resistance to bacterial infections such as *Listeria monocytogenes* and *Yersinia pestis* by activating the host immune response (27). Similarly, bacteriophage plays an important role in regulating bacterial population in the host and participating in immune system development, and maintaining immune homeostasis in the body organ such as in the intestine (28–30).

## Long-term interactions between the virus and its host left traces in the host genome

The genome of humans and animals encodes from a few to 100,000 different genes (31, 32). It is estimated that around 8% or around one-tenth of the human gene contains pieces of viral DNA (33). These viral DNA fragments incorporated in the host is termed as endogenous viral elements (EVEs) (34). EVEs provide valuable information about their evolution over the time, their host, and geographical distribution (35, 36).

Integration of viral DNA into the host genome is not always harmful. Evidence showed that the presence of EVEs provides antiviral defense against viral infection by encoding several genes such as ribonuclease H (RNase H) or small interfering RNAs (siRNAs) which silence the viral gene in subsequent infection (35, 37, 38). Interestingly, several EVEs such as the *Arc* gene, play an important role in cognitive function and help to store long-term memory in the host (39, 40). Another study revealed that host cells can use EVEs to induce immunity against tumors (41). Cellular p53 is a nuclear transcription factor with pro-apoptotic function. Cellular stresses such as DNA damage activates p53 and activated p53 promotes cell cycle arrest that allows time for DNA repair or causes apoptosis in the damaged cells (42). Studies showed that p53 activation also increased the expression of EVEs and EVE's expression induced host immune response in the form of higher IFN production, increased T-cell activity, and reduced allograft tumor (41). However, the presence of EVEs is not always beneficial to the host. Studies showed that individuals with inherited chromosomally integrated human herpesvirus 6 (iciHHV-6) gene are more susceptible to angina, a type of chest pain that happens when heart does not receive enough oxygen-rich blood (43).

## Host-virus interaction and its outcome

Translocation of virus particles to the host cell is the first step in virus infection. This translocation is initiated by the interaction of distinct molecules present on the outer layer of the virus with specific receptors on the host cell (44). This initial interaction provides signals to the cells which further enhance the virus translocation to the host cell either by receptor-mediated endocytosis or membrane fusion, these mechanism varies widely among different virus families (45, 46). In the host cell, virus releases its genetic material for replication by fusing the viral capsid with the host cell's phagosome or creating pores in the viral capsid by utilizing cellular or viral machinery (47, 48). During this process host immune system recognize the virus by its pattern recognition receptors (PRRs) which specifically interact with pathogen-associated molecular patterns (PAMPs) present in the virus. This recognition triggers a cascade of innate immune responses, including the secretion of interferons/cytokines and the activation of immune cells (49). The outcome of this host–viral interaction depends on several host or viral factors (50). These outcomes can result in the clearing of virus by host immune response (51–53), or host immune evasion by virus (54), or establishing acute, chronic, persistent, or latent infection (55–57). It is found that a high mutation rate in viruses helps them to evolve, survive and escape from the host's immune response. While these changes may also be responsible for shifting in virus host range (58–60).

## Damage in viral disease

The outcome of viral disease is greatly influenced by viral factors such as virulence and host factors like susceptibility. Broadly, damage in the host during virus infection can be divided into two, (A) direct damage by the virus when virus replicates in the host cell and cause damage in cells either by hijacking cellular machinery, changing cellular physiology, or damaging cellular components by its structural or non-structural proteins (61–63). (B) The virus can also cause indirect damage to the host cell by virus-induced hyperinflammation (64, 65). Virus-induced uncontrol-hyperinflammation has been associated with higher mortality in several virus infections (66–68).

## Cellular factors in restricting virus replication

Host cells have several inherent antiviral factors such as antiviral proteins which control virus replication. A few of the antiviral proteins are APOBEC3G (Apolipoprotein B mRNA Editing Enzyme, Catalytic Polypeptide-like 3G), ZAP (Zinc Finger Antiviral Protein), SAMHD1 (SAM domain and HD domain-containing protein 1), 2′-5′-Oligoadenylate Synthetase (OAS), Mx Proteins and Tetherin, which is also known as BST-2 (bone marrow stromal antigen 2) (69–75).

APOBEC3G exhibits its antiviral effect by inducing hypermutation in viral genomes. APOBEC3G catalyzes the deamination of cytosine (dC) to uracil (dU) in single-stranded DNA, leading to non-functional viral DNA and prevents virus replication (76). While ZAP degrades the viral RNA by binding with its poly A tail. This binding leads to deadenylation of viral RNA and viral RNA is further degraded by exosomes (77). ZAP also selectively recognizes CG-rich viral RNAs and degrades viral RNA by exosomes (70). Additionally, ZAP could suppress virus replication by inducing an antiviral immune response in the host (78).

SAMHD1, another cellular protein exhibits its antiviral effect by hydrolyzing nucleotide triphosphates (dNTPs), dNTPs are needed by the virus to synthesize its genome while the lower concentration of dNTP in cells suppress the virus replication (79). However, a recent study also showed that SAMHD1 can suppress the antiviral host immune response by inhibiting NF-κB (Nuclear factor kappa-light-chain-enhancer of activated B cells) activation, suppressing IFN-I (type-I interferons) response and potentially interfering in virus-specific antibody production (80).

2′-5′-Oligoadenylate Synthetase (OAS), activates RNase L, an endoribonuclease that cleaves single-stranded viral RNAs and suppresses its replication (81), while Mx proteins, upon activation, interact with viral nucleocapsid proteins and disrupts viral replication complexes and thus inhibits the viral life cycle (82). Tetherin, on the other hand, exhibits its antiviral effect by physically “tethering” newly synthesized virus and present its release (83). Above mentioned proteins are a few of the cellular proteins which play an important role in suppressing virus replication. Potentially, there may be numerous more cellular molecules including small interfering RNA (siRNA) which suppress virus replication in the host cell. These molecules need to be studied in detail to understand the underlying pathways and their role in host-virus interactions in suppressing virus replication.

## Summary

Host-virus interaction studies provide valuable information about viruses, and their molecular characteristics which facilitate them to mutate, change their virulence, and shift host range. Additionally, these studies help in understanding the effect of virus infection on host cells, cell organelles, and the physiological/metabolic activity of the cells. These studies also help in identifying the cellular molecules which could have antiviral properties. These molecules could be used to suppress the broad range of pathogenic viruses.

## Author contributions

MR: Writing—original draft, Writing—review and editing. NT: Writing—original draft, Writing—review and editing.
